# Association between annual air pollutant exposure and thyroid function: a retrospective cross-sectional study

**DOI:** 10.1007/s40618-026-02965-6

**Published:** 2026-08-29

**Authors:** Sinyoung Cho, Seo Eun Hwang, Jae Moon Yun, Soon Tae Kim, Yoon Hee Kang, Hyun-Jin Kim, Jin-Ho Park

**Affiliations:** 1 Department of Family Medicine, Seoul National University Hospital, Seoul, South Korea; 2 Department of Family Medicine, Seoul National University College of Medicine, Seoul, South Korea; 3 Department of Environmental and Safety Engineering, Ajou University, Suwon-si, South Korea; 4 Environmental Institute, Ajou University, Suwon-si, South Korea; 5 Cancer Big Data Center, National Cancer Control Institute, National Cancer Center, Goyang-si, South Korea; 6 103 Daehakro, Yeongun-dong, Jongno-gu, Seoul, 03080 South Korea; 7 323 Ilsan-ro, Ilsandong-gu, Goyang-si, 10408 Gyeonggi-do South Korea

**Keywords:** Air pollution, Ambient particulate matter, Thyroid function test, Thyrotropin, Thyroxine

## Abstract

**Aims:**

Despite the high burden of air pollutants, the association between air pollutant exposures and thyroid function in the general population is inconclusive. This study aimed to investigate the association between annual exposure to air pollutants and thyroid function in Korean adults.

**Methods:**

This cross-sectional study included a total of 16,193 participants (8,812 males, 7,381 females; mean age: 53.8 ± 13.1 years) who underwent health examinations at Seoul National University and had thyroid-stimulating hormone (TSH) concentrations within the range of 0.1–10.0 mIU/L. The yearly average levels of five air pollutants, including particulate matter (PM_2.5_ and PM_10_), NO_2_, SO_2_, and CO, were estimated using the Community Multiscale Air Quality model. Multivariable linear and logistic regression models were used to examine the associations between annual ambient air pollutant exposures and thyroid function parameters, including TSH and free thyroxine (FT4).

**Results:**

Interquartile range (IQR) increases for PM_2.5_, PM_10_, NO_2_, SO_2_, and CO were positively associated with TSH and negatively associated with FT4 (all *P* < 0.001). Significant non-linear associations were observed for PM_2.5_ and NO₂ with log-transformed TSH, whereas all air pollutants showed non-linear associations with log-transformed FT4. The IQR increases of all air pollutants showed positive associations with the highest quartile of TSH (high TSH) and the lowest quartile of FT4 (low FT4), respectively. When thyroid function was categorized into four groups based on high/low TSH and/or high/low FT4, significantly positive associations were demonstrated with high TSH–low FT4 group, with the strongest association observed for PM_10_ (adjusted odds ratio [aOR], 1.55; 95% confidence interval [CI], 1.42–1.69), followed by SO_2_ (1.38, 1.28–1.49), NO_2_ (1.36,1.23–1.50), PM_2.5_ (1.25, 1.17–1.34), and CO (1.23, 1.15–1.31). The sex-stratified analyses demonstrated more prominent associations among females.

**Conclusions:**

Yearly exposure levels of air pollutants were positively associated with TSH, negatively associated with FT4 levels, and significantly associated with high TSH-low FT4. These findings suggest that exposure to ambient air pollution may contribute to thyroid function alterations at the population level, highlighting the importance of air quality improvement. Further longitudinal studies were warranted to consolidate these findings.

**Supplementary Information:**

The online version contains supplementary material available at https://doi.org/10.1007/s40618-026-02965-6.

## Introduction

Air pollution remains one of the leading global environmental health risks, contributing to an estimated 8.1 million deaths worldwide in 2021 [1]. Exceeding 90% of the global population is exposed to levels above World Health Organization (WHO) air quality guidelines for annual air pollutant exposures [2]. Elevated air pollutant concentrations in South Korea arise from local traffic emissions, industrial activities, and Asian dust, a seasonal phenomenon characterized by the long-range transport of mineral dust particles from arid regions of East Asia [3]. In South Korea, despite the implementation of national air pollution monitoring, the annual average level of particulate matter with diameters of ≤ 2.5 μm (PM_2.5_) remained elevated at 19 µg/m³ in 2023, exceeding the WHO guideline (5 µg/m³) [2, 4]. Previous literature has demonstrated that both short-term and long-term exposure to air pollution, despite heterogeneous operational definitions, is associated with a broad range of adverse health outcomes, including cardiovascular disease [5], cognitive impairment [6], depression [7], and increased mortality [8].

Thyroid disorders are among the most prevalent endocrine disorders, affecting approximately 200 million individuals worldwide [9, 10]. Thyroid dysfunction, encompassing both subclinical and overt forms, has been implicated in health outcomes, particularly cardiovascular mortality [11]. Moreover, even subtle changes in thyroid function within the reference range have been associated with low muscle mass [12], atrial fibrillation [13], and depression [14]. Recent studies have reported potential associations between air pollutant exposures and alterations in thyroid function; however, the associations remain inconsistent [15–17]. Growing interest in this field has led to a recent meta-analysis of the association between ambient air pollution and thyroid function, which analyzed 14 studies, including 7 conducted among pregnant women, 2 among newborns, 2 encompassing both groups, and 3 involving general adult populations [16]. The meta-analysis encompassing 357,226 participants reported significant associations between exposure to PM_2.5_, PM_10_, NO_2_, SO_2_, and CO and reductions in free thyroxine (FT4), whereas only PM_2.5_ showed a significant association with elevated thyroid-stimulating hormone (TSH).

Despite an increasing number of epidemiological studies, the studies for general populations, on PM_2.5_, and utilizing modeling-based air pollution estimation methods with higher accuracy, are still very limited. Therefore, the aim of this study was to investigate the association between annual average levels of air pollutant exposure, estimated using refined and accurate modeling methods, and thyroid function in a large cohort. Additionally, we examined whether these associations differed across stratified subgroups based on lifestyle factors.

## Methods

### Study population and data source

This cross-sectional study utilized health examination data collected from Seoul National University Hospital Health Promotion Center between January 1, 2015, and December 31, 2019. A total of 19,159 adults underwent health checkups during the study period. We excluded participants with self-reported prior diagnosis of thyroid disease, including hyperthyroidism, hypothyroidism, and thyroid cancer (*n* = 551), and those with missing information on covariates (*n* = 2,230). We included individuals with serum TSH levels ranging from 0.1 to 10.0 mIU/L because most guidelines suggest that TSH levels below 0.1 mIU/L or above 10 mIU/L are associated with autoimmune or toxic nodular goiter rather than environmental causes [18]. In addition, subjects without residential address information (city, county, or district) for matching air pollution concentrations were excluded (*n* = 130). Therefore, a total of 16,193 participants were included in the final analysis (Supplementary Fig. 1). This study was approved by the Institutional Review Board of Seoul National University Hospital (IRB No. H-2202-073-1299) and conducted in accordance with the principles of the Declaration of Helsinki (revised 2013). The requirement for informed consent was waived by the committee due to the retrospective design and use of anonymized data.

### Thyroid function

Thyroid function was evaluated by serum TSH (mIU/L) and FT4 (ng/dL), which were quantified utilizing Chemiluminescent Microparticle Immuno Assay, and the same assay was applied throughout the study. The assay-specific reference ranges for TSH were 0.35–<4.94 uIU/mL, and FT4 was 0.70–<1.48 ng/dL. Although the diagnosis of hypo- and hyperthyroidism is based on abnormal levels of assay-specific reference ranges, the cut-off values used to define thyroid dysfunction vary across clinical guidelines and treatment thresholds [19–21]. For this reason, we first categorized serum TSH into sex-specific quartiles based on the distribution within the study population. The quartile cut-offs of TSH were as follows: for male, < 1.12, 1.12–<1.44, 1.44–<1.96, and ≥ 1.96 mIU/L; for female, < 1.22, 1.22–<1.56, 1.56–<2.26, and ≥ 2.26 mIU/L. Serum FT4 was categorized with the following cutoffs: for males, < 1.18, 1.18–<1.29, 1.29–<1.40, and ≥ 1.40 ng/dL; for females, < 1.14, 1.14–<1.24, 1.24–<1.34, and ≥ 1.34 ng/dL. Serum FT4 was evaluated independently of TSH, given its potential to provide prognostic information on thyroid dysfunction even on its own [22]. Binary outcomes were first defined as high TSH with TSH levels above the 75th percentile (1.96 mIU/L for males; 2.26 mIU/L for females) and low FT4 with FT4 below the 25th percentile (1.18 mIU/L for males; 1.14 mIU/L for females). Additionally, thyroid function was categorized into four groups based on high/low TSH (above the 75th percentile/below the 75th percentile) and/or high/low FT (above the 25th percentile/below the 25th percentile). These categories were defined based on biochemical measurements rather than clinical diagnoses.

### Air pollution data

Annual air pollution exposure levels were estimated using hourly concentration data obtained from more than 300 nationwide regulatory air quality monitoring stations in Korea, operated by the Ministry of Environment. This study included five air pollutants, comprising PM_2.5_ and particulate matter with diameters of ≤ 10 μm (PM₁₀), as well as gaseous pollutants, including NO₂, sulfur dioxide (SO₂), and carbon monoxide (CO). The modeling methods utilized the Community Multiscale Air Quality (CMAQ) model version 4.7.1, which was developed by the US Environmental Protection Agency [23]. Meteorological and emission datasets were incorporated into the CMAQ through the Weather Research and Forecasting (WRF, version 3.9) and Sparse Matrix Operator Kernel Emissions (SMOKE, version 3.1) models. The simulation employed a horizontal grid with a 9-km spatial resolution, enabling fine-scale geographic analysis. The details of the CMAQ model have been described in detail in previous studies [24–27]. Concentrations for each air pollutant were linked to 250 administrative district (city, county, district) codes in South Korea. Participants` residential addresses were subsequently converted to corresponding administrative district codes and linked to annual average air pollution concentrations estimated for lag day 0 (date of examination) to lag day 365 (365 days before the date of examination) as a proxy for annual exposure.

### Covariates

Smoking status, alcohol consumption, and physical activity were assessed using a self-reported questionnaire collected on the same day during health checkups. Smoking status was classified into three categories: never smoked, former, and current smoker. Daily alcohol intake was estimated by multiplying the number of drinks consumed per week by the standard drink size and dividing the total by seven days. Alcohol consumption was categorized into three groups: non-drinkers, individuals consuming fewer than two drinks per day, and those consuming two or more drinks per day. Weekly physical activity was measured in metabolic equivalent (MET) minutes per week. The International Physical Activity Questionnaire (IPAQ) scoring protocol was utilized to assess the duration and frequency of walking and other moderate-to-vigorous physical activities throughout seven-day periods [28]. Further categorization of MET minutes per week was applied based on the recommendations of the Centers for Disease Control and Prevention [29]. Diabetes mellitus was defined as a fasting glucose level of ≥ 126 mg/dL, hemoglobin A1c (HbA1c) of ≥ 6.5%, or the use of glucose-lowering medication. Hypertension was defined as a systolic blood pressure (BP) of ≥ 140 mmHg, a diastolic BP of ≥ 90 mmHg, or the use of BP-lowering medication. Dyslipidemia was defined as a low-density lipoprotein (LDL) cholesterol level of ≥ 130 mg/dL or the use of lipid-lowering medication. Additionally, environmental factors were taken into account, including the season at the time of the health examination (spring, summer, fall, or winter) and the average temperature on the day of examination.

### Statistical analyses

Before analysis, the distribution of thyroid hormone levels was assessed for normality. As they did not follow a normal distribution, their values were log-transformed using the natural logarithm to approximate normality. Comparisons between sexes were conducted using a student’s t-test for continuous variables and the chi-squared test for categorical variables. For comparisons involving more than two groups, post-hoc analyses were conducted using adjusted residuals to identify significant pairwise differences. For the selection of covariates, we performed the univariate linear regression analysis for thyroid function (Supplementary Table 1). Significant predictors with a *P*-value < 0.20 were selected as final covariates [30]. Additionally, dyslipidemia was considered a potential confounder from a previously reported association with thyroid function [31]. There was no multicollinearity among variables in the model (variance inflation factor < 5).

Linear regression models were utilized to examine the associations between annual average air pollution concentrations and thyroid function in both crude and adjusted models. To assess potential non-linear associations between air pollutant exposure and thyroid function, restricted cubic spline models were applied. Logistic regression analyses were additionally performed to assess the associations of air pollution exposure with thyroid hormone parameters in quartile-based comparisons (TSH: Q4 vs. Q1–3; FT4: Q1 vs. Q2–4). Multinomial logistic regression analyses were used for four categories of thyroid function, including low TSH/high FT4, high TSH/high FT4, low TSH/low FT4, and high TSH/low FT4. Sensitivity analyses were performed after excluding participants with low TSH /high FT4 to account for potential overt hyperthyroidism.

The adjusted models accounted for potential confounders, including sex (categorical, for total sample only), age (continuous), BMI (continuous), smoking status (categorical), daily alcohol intake (categorical), physical activity (categorical), diabetes mellitus (categorical), hypertension (categorical), dyslipidemia (categorical), season (categorical), and temperature (continuous). The estimates from the linear and logistic regression models were scaled to reflect changes per interquartile range (IQR) increase for each air pollutant. Further stratified analyses were conducted for quartile analysis to assess differences in the associations according to median age, BMI of 25 kg/m^2^, METs minutes per week (1,000 METs), alcohol intake (none or more than one drink per day), and smoking status (never or at least 100 cigarettes during lifetime). *P* values for interaction were calculated using the likelihood ratio test.

All analyses were performed utilizing R software (version 4.1.2), with statistical significance defined at *p* < 0.05.

## Results

### Descriptive characteristics

Table 1 presents the baseline characteristics of a total of 16,193 participants (male 54.4%, female 45.6%) according to sex. The mean age of the total sample was 53.8 ± 13.1 years. The proportions of patients with hypertension and diabetes mellitus were 32.6% and 13.1%, respectively, and more than half of the subjects had dyslipidemia (51.1%). In the case of thyroid hormones, the mean values of TSH and FT4 in the total sample were 1.8 mIU/L and 1.3 ng/dL, respectively. For ambient air pollutants, the mean annual concentrations of PM_2.5_, PM_10_, NO_2_, SO_2_, and CO were 24.6 µg/m^3^, 45.5 µg/m^3^, 27.3 ppb, 4.4 ppb, and 509.3 ppb, respectively, and the IQR for PM_2.5_, PM_10_, NO_2_, SO_2_, and CO were 2.7 µg/m^3^, 7.4 µg/m^3^, 14.1 ppb, 1.2 ppb, and 83.5 ppb, respectively.

**Table 1:** General characteristics of participants

	Total	Male	Female	*p*-value
	*N* (%) or mean (SD)	*N* (%) or mean (SD)	*N* (%) or mean (SD)	
Total, No.	16,193	8,812 (54.4%)	7,381 (45.6%)	
Age (years)	53.8 (13.1)	54.2 (13.1)	53.4 (13.0)	< 0.001
TSH (mIU/L)	1.8 (1.1)	1.7 (1.0)	1.9 (1.2)	< 0.001
FT4 (ng/dL)	1.3 (0.2)	1.3 (0.17)	1.2 (0.16)	< 0.001
Temperature (°C)	12.9 (10.7)	12.8 (10.7)	13.0 (10.8)	0.234
BMI (kg/m^2^)	23.60 (3.4)	24.49 (3.2)	22.55 (3.4)	< 0.001
*BMI group (kg/m* ^*2*^ *)*				< 0.001
< 18.5	810 (5.0)	163 (1.8)*	647 (8.8)*	
18.5 ≤ BMI < 23	6,387 (39.4)	2,625 (29.8)*	3,762 (51.0)*	
23 ≤ BMI < 25	4,007 (24.7)	2,519 (28.6)*	1,488 (20.2)*	
BMI ≥ 25	4,989 (30.8)	3,505 (39.8)*	1,484 (20.1)*	
Physical activity (MET minutes per week)				< 0.001
METs < 500	6,331 (39.1)	3,189 (36.2)*	3,142 (42.6)*	
500 < METs < 1000	2,118 (13.1)	1,107 (12.6)*	1,011 (13.7)*	
METs ≥ 1000	7,744 (47.8)	4,516 (51.2)*	3,228 (43.7)*	
*Alcohol intake*				< 0.001
None	6,711 (42.9)	2,205 (26.0)*	4,506 (62.9)*	
Less than 2 drinks/day	6,376 (40.7)	3,944 (46.5)*	2,432 (33.9)*	
More than 2 drinks/day	2,569 (16.4)	2,341 (27.6)*	2,28 (3.2)*	
*Smoking habit*				< 0.001
Never smoker	8,882 (54.9)	2,167 (24.6)*	6,715 (91.0)*	
Ex-smoker	4,316 (26.7)	3,963 (45.0)*	353 (4.8)*	
Current smoker	2,995 (18.5)	2,682 (30.4)*	313 (4.2)*	
Hypertension	5,282 (32.6)	3,396 (38.5)	1,886 (25.6)	< 0.001
Diabetes mellitus	2,118 (13.1)	1,453 (16.5)	665 (9.0)	< 0.001
Dyslipidemia	8,273 (51.1)	4,642 (52.7)	3,631 (49.2)	< 0.001
*Season*				< 0.001
Spring	3,925 (24.2)	2,133 (24.2)	1,792 (24.3)	
Summer	4,153 (25.6)	2,258 (25.6)	1,895 (25.7)	
Autumn	3,931 (24.3)	2,133 (24.2)	1,798 (24.4)	
Winter	4,184 (25.8)	2,288 (26.0)	1,896 (25.7)	
*Air pollutants*				
PM_2.5_ (µg/m^3^)	24.6 (2.5)	24.6 (2.5)	24.7 (2.4)	0.212
PM_10_ (µg/m^3^)	45.5 (5.4)	45.4 (5.5)	45.6 (5.3)	0.071
NO_2_ (ppb)	27.3 (8.9)	27.0 (9.1)	27.7 (8.8)	< 0.001
SO_2_ (ppb)	4.4 (0.9)	4.4 (1.0)	4.4 (0.9)	0.003
CO (ppb)	509.3 (71.1)	507.2 (72.8)	511.9 (69.0)	< 0.001

Data are presented as mean ± SD for continuous variables, or No. (%) for categorical variables

TSH, thyrotropin; FT4, free thyroxine; BMI, body mass index; METs, Metabolic Equivalent Task minutes per week; PM_2.5_, particulate matter ≤ 2.5 μm in diameter; PM_10_, particulate matter ≤ 10 μm in diameter; NO_2_, nitrogen dioxide; SO_2_, sulfur dioxide; CO, carbon monoxide; SD, standard deviations

The P-value was calculated by Chi-squared test for categorical variables and Student’s t-test for continuous variable. Adjusted residuals were calculated for post-hoc comparisons following chi-square tests. Values exceeding |1.96| were considered statistically significant. Statistically significant cells are indicated with an asterisk ()*

### Annual air pollutant exposures and thyroid function

**Fig. 1:**
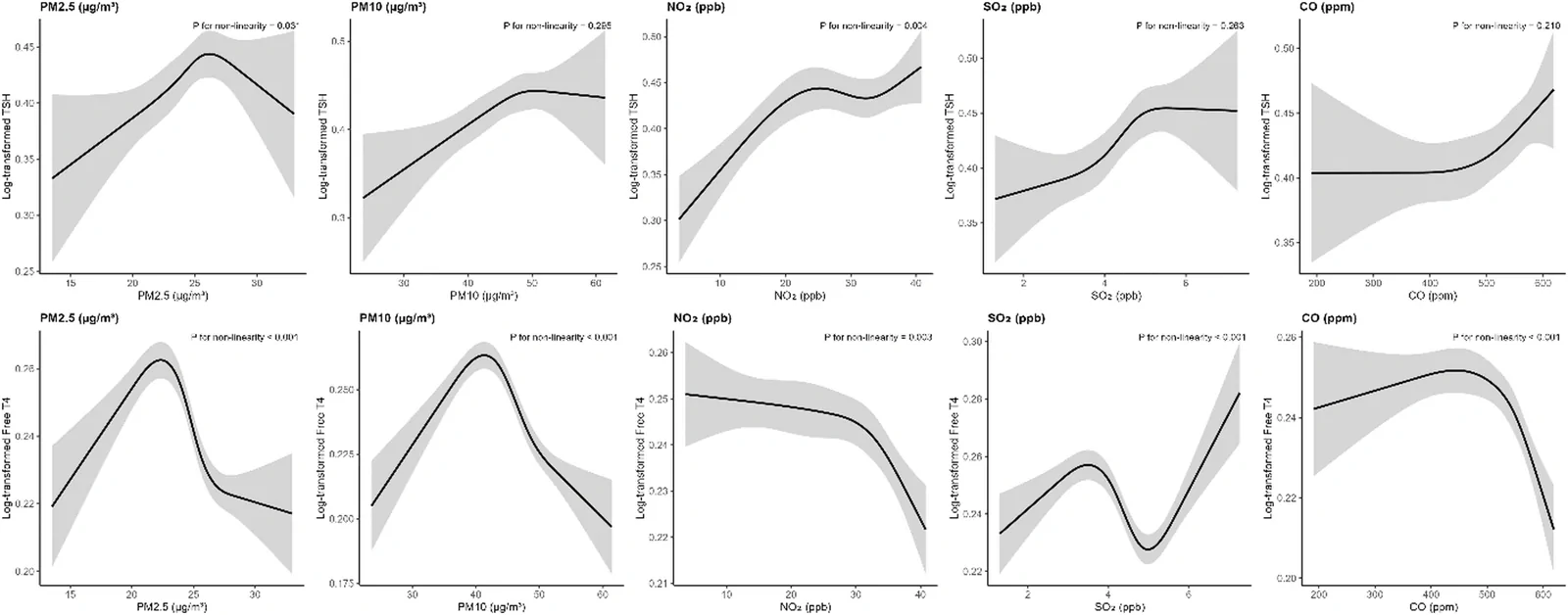
Association of annual exposure to air pollution with log-transformed TSH and log-transformed FT4 TSH, thyrotropin; FT4, free thyroxine; PM_2.5_, particulate matter ≤ 2.5 μm in diameter; PM_10_, particulate matter ≤ 10 μm in diameter; NO_2_, nitrogen dioxide; SO_2_, sulfur dioxide; CO, carbon monoxide. Hazard ratios are indicated by solid lines and 95% CIs by shaded areas. Data were adjusted for sex (categorical), age (continuous), BMI (continuous), smoking status (categorical), daily alcohol intake (categorical), physical activity (categorical), diabetes mellitus (categorical), hypertension (categorical), dyslipidemia (categorical), season (categorical), and temperature (continuous)

In linear regression analysis, all five air pollutants showed positive associations with log-transformed TSH level (all *p* < 0.05) and negative associations with log-transformed FT4 level (all *p* < 0.05) in both crude and adjusted models (Table 2). In the sex-stratified analysis, associations between air pollution exposure and log-transformed TSH or log-transformed FT4 level were similar between the two groups. However, the point estimates for each IQR increase in all air pollutants were relatively greater in females than in males. Interestingly, in males, PM_2.5_, PM_10_, and CO did not show significant associations with log-transformed TSH in the unadjusted model, but after adjusting for covariates, significant associations with log-transformed TSH were observed in PM_10_ and CO. In the case of log-transformed FT4, exposures to NO_2_, SO_2_, and CO were not significant in the unadjusted model, but all three pollutants showed significant associations in the adjusted model among males (Table 2). In restricted cubic spline analyses, the evidence of non-linearity was observed for PM_2.5_ (*p* for non-linearity = 0.031) and NO₂ (*p* for non-linearity = 0.004), whereas the associations for PM_10_, SO₂, and CO were consistent with linear trends (all *p* for non-linearity > 0.05) (Fig. 1). For log-transformed FT4, PM_2.5_ and PM_10_ demonstrated significant non-linear associations (both *p* for non-linearity < 0.001), characterized by increasing FT4 levels at lower exposure ranges followed by a decline at higher concentrations. NO₂ also showed a non-linear association (*p* for non-linearity = 0.003). Similarly, SO₂ and CO exhibited significant non-linear relationships (both *p* for non-linearity < 0.001), with heterogeneous patterns across exposure levels.

**Table 2:** Linear regression of the association of annual exposure to air pollution with log-transformed TSH and log-transformed FT4

Air pollutants exposures	Total (*n* = 16,193)				Male (*n* = 8,812)				Female (*n* = 7,381)			
	Unadjusted		Adjusted		Unadjusted		Adjusted		Unadjusted		Adjusted	
	β (SE)	*p*-value	β (SE)	*p*-value	β (SE)	*p*-value	β (SE)	*p*-value	β (SE)	*p*-value	β (SE)	*p*-value
*Log TSH*												
PM_2.5_ (µg/m^3^)	**0.015 (0.005)**	**0.002**	**0.016 (0.005)**	**0.001**	0.009 (0.006)	0.133	0.011 (0.006)	0.084	**0.021 (0.007)**	**0.004**	**0.024 (0.007)**	**0.001**
PM_10_ (µg/m^3^)	**0.024 (0.006)**	**< 0.001**	**0.026 (0.006)**	**< 0.001**	0.014 (0.008)	0.071	**0.017 (0.008)**	**0.034**	**0.034 (0.009)**	**< 0.001**	**0.039 (0.009)**	**< 0.001**
NO_2_ (ppb)	**0.036 (0.007)**	**< 0.001**	**0.039 (0.007)**	**< 0.001**	**0.032 (0.009)**	**0.001**	**0.033 (0.009)**	**< 0.001**	**0.036 (0.011)**	**0.001**	**0.044** **(0.011)**	**<0.001**
SO_2_ (ppb)	**0.027 (0.006)**	**< 0.001**	**0.029 (0.006)**	**< 0.001**	**0.025 (0.007)**	**0.001**	**0.026 (0.007)**	**< 0.001**	**0.028 (0.008)**	**0.001**	**0.034 (0.008)**	**< 0.001**
CO (ppb)	**0.015 (0.005)**	**0.003**	**0.017 (0.005)**	**0.001**	0.011 (0.007)	0.103	**0.014 (0.007)**	**0.042**	**0.017 (0.008)**	**0.033**	**0.022 (0.008)**	**0.006**
*Log FT4*												
PM_2.5_ (µg/m^3^)	**− 0.009 (0.001)**	**< 0.001**	**− 0.011 (0.001)**	**< 0.001**	**-0.008 (0.002)**	**< 0.001**	**− 0.010 (0.002)**	**< 0.001**	**− 0.011 (0.002)**	**< 0.001**	**− 0.012 (0.002)**	**< 0.001**
PM_10_ (µg/m^3^)	**− 0.013 (0.001)**	**< 0.001**	**− 0.016 (0.001)**	**< 0.001**	**− 0.008 (0.002)**	**< 0.001**	**− 0.012 (0.002)**	**< 0.001**	**− 0.018 (0.002)**	**< 0.001**	**− 0.21 (0.002)**	**< 0.001**
NO_2_ (ppb)	**− 0.006 (0.002)**	**0.001**	**− 0.009 (0.002)**	**< 0.001**	**−** 0.001 (0.002)	0.682	**− 0.006 (0.002)**	**0.007**	**− 0.009 (0.002)**	**< 0.001**	**− 0.013 (0.003)**	**< 0.001**
SO_2_ (ppb)	**− 0.005 (0.001)**	**< 0.001**	**− 0.007 (0.001)**	**< 0.001**	**−** 0.001 (0.002)	0.749	**− 0.004 (0.002)**	**0.028**	**− 0.009 (0.002)**	**< 0.001**	**− 0.011 (0.002)**	**< 0.001**
CO (ppb)	**− 0.005 (0.001)**	**< 0.001**	**− 0.007 (0.001)**	**< 0.001**	**−** 0.002 (0.002)	0.306	**− 0.005 (0.002)**	**0.001**	**− 0.007 (0.002)**	**< 0.001**	**− 0.010 (0.002)**	**< 0.001**

TSH, thyrotropin; FT4, free thyroxine; PM_2.5_, particulate matter ≤ 2.5 μm in diameter; PM_10_, particulate matter ≤ 10 μm in diameter; NO_2_, nitrogen dioxide; SO_2_, sulfur dioxide; CO, carbon monoxide; SE, standard error

The beta coefficients (SE) of air pollutants were scaled to the interquartile range (IQR) for each air pollutant: 2.7 µg/m^3^ for PM_2.5_, 7.4 µg/m^3^ for PM_10_, 14.1 ppb for NO_2_, 1.2 ppb for SO_2_ and 83.5 ppb for CO

In the adjusted model, data were adjusted for sex (categorical, for total sample only), age (continuous), BMI (continuous), smoking status (categorical), daily alcohol intake (categorical), physical activity (categorical), diabetes mellitus (categorical), hypertension (categorical), dyslipidemia (categorical), season (categorical), and temperature (continuous).

Bold values indicate statistically significant associations (P < 0.05).

In both crude and adjusted logistic regression models, all air pollutants, including PM_2.5_, PM_10_, NO_2_, SO_2_, and CO, were positively associated with the highest quartile of TSH and the lowest quartile of FT4 (all *p* < 0.05), respectively (Table 3). These associations also remained after stratification by sex, except NO_2_ with the lowest quartile FT4 in males and CO with the highest quartile TSH in females. In the total sample, the point estimates of the lowest quartile FT4 group for each IQR increase were most pronounced for PM_10_ exposure (adjusted odds ratio = 1.40; 95% CI = 1.33–1.47). Notably, PM_10_ exposure in females had the highest OR for the lowest quartile FT4 (adjusted OR = 1.51; 95% CI = 1.39–1.64).

**Table 3:** Multivariable logistic regression of the associations between annual air pollution exposure and quartile-based dichotomized TSH and FT4 Levels

Air pollutant exposures	Total (*n* = 16,193)				Male (*n* = 8,812)				Female (*n* = 7,381)			
	Unadjusted		Adjusted		Unadjusted		Adjusted		Unadjusted		Adjusted	
	OR (95% CI)	*p*	OR (95% CI)	*p*	OR (95% CI)	*p*	OR (95% CI)	*p*	OR (95% CI)	*p*	OR (95% CI)	*p*
*High TSH (Quartile 4 versus Quartile 1–3)*												
PM_2.5_ (µg/m^3^)	**1.07 (1.03**,** 1.11)**	**0.001**	**1.07 (1.03**,** 1.12)**	**< 0.001**	**1.07 (1.01**,** 1.13)**	**0.013**	**1.08 (1.02**,** 1.13)**	**0.008**	**1.06 (1.00**,** 1.13)**	**0.039**	**1.08 (1.01**,** 1.14)**	**0.016**
PM_10_ (µg/m^3^)	**1.11 (1.06**,** 1.17)**	**< 0.001**	**1.12 (1.07**,** 1.18)**	**< 0.001**	**1.11 (1.04**,** 1.19)**	**0.001**	**1.13 (1.05**,** 1.21)**	**0.001**	**1.10 (1.03**,** 1.19)**	**0.009**	**1.12 (1.04**,** 1.21)**	**0.003**
NO_2_ (ppb)	**1.11 (1.05**,** 1.17)**	**0.001**	**1.13 (1.07**,** 1.20)**	**< 0.001**	**1.14 (1.05**,** 1.23)**	**0.001**	**1.16 (1.08**,** 1.26)**	**< 0.001**	1.07 (0.98, 1.17)	0.117	**1.10 (1.01**,** 1.20)**	**0.033**
SO_2_ (ppb)	**1.11 (1.06**,** 1.16)**	**< 0.001**	**1.13 (1.08**,** 1.18)**	**< 0.001**	**1.11 (1.05**,** 1.18)**	**0.001**	**1.13 (1.06**,** 1.20)**	**< 0.001**	**1.11 (1.04**,** 1.19)**	**0.003**	**1.13 (1.06**,** 1.21)**	**0.001**
CO (ppb)	**1.04 (1.00**,** 1.09)**	**0.042**	**1.06 (1.01**,** 1.11)**	**0.010**	1.05 (0.99, 1.11)	0.079	**1.07 (1.01**,** 1.13)**	**0.024**	1.04 (0.97, 1.11)	0.278	1.05 (0.99, 1.12)	0.132
*Low FT4 (Quartile 1 versus Quartiles 2–4)*												
PM_2.5_ (µg/m^3^)	**1.16 (1.11**,** 1.20)**	**< 0.001**	**1.18 (1.14**,** 1.23)**	**< 0.001**	**1.13 (1.07**,** 1.19)**	**< 0.001**	**1.16 (1.10**,** 1.23)**	**< 0.001**	**1.19 (1.12**,** 1.26)**	**< 0.001**	**1.21 (1.14**,** 1.29)**	**< 0.001**
PM_10_ (µg/m^3^)	**1.34 (1.28**,** 1.41)**	**< 0.001**	**1.40 (1.33**,** 1.47)**	**< 0.001**	**1.25 (1.17**,** 1.34)**	**< 0.001**	**1.32 (1.23**,** 1.42)**	**< 0.001**	**1.47 (1.36**,** 1.59)**	**< 0.001**	**1.51 (1.39**,** 1.64)**	**< 0.001**
NO_2_ (ppb)	**1.08 (1.02**,** 1.14)**	**0.012**	**1.13 (1.07**,** 1.20)**	**< 0.001**	1.00 (0.93, 1.08)	0.932	1.07 (0.99, 1.15)	0.105	**1.18 (1.08**,** 1.29)**	**< 0.001**	**1.23 (1.12**,** 1.34)**	**< 0.001**
SO_2_ (ppb)	**1.17 (1.11**,** 1.22)**	**< 0.001**	**1.20 (1.15**,** 1.26)**	**< 0.001**	**1.09 (1.03**,** 1.16)**	**0.005**	**1.14 (1.07**,** 1.22)**	**< 0.001**	**1.27 (1.18**,** 1.36)**	**< 0.001**	**1.29 (1.20**,** 1.38)**	**< 0.001**
CO (ppb)	**1.06 (1.01**,** 1.10)**	**0.010**	**1.10 (1.05**,** 1.15)**	**< 0.001**	1.03 (0.97, 1.09)	0.353	**1.07 (1.01**,** 1.14)**	**0.015**	**1.10 (1.03**,** 1.18)**	**0.004**	**1.14 (1.06**,** 1.22)**	**< 0.001**

TSH, thyrotropin; FT4, free thyroxine; PM_2.5_, particulate matter ≤ 2.5 μm in diameter; PM_10_, particulate matter ≤ 10 μm in diameter; NO_2_, nitrogen dioxide; SO_2_, sulfur dioxide; CO, carbon monoxide; OR, odds ratio; CI, confidence interval

The ORs and 95% CIs of air pollutants were scaled to the interquartile range (IQR) for each air pollutant: 2.7 µg/m^3^ for PM_2.5_, 7.4 µg/m^3^ for PM_10_, 14.1 ppb for NO_2_, 1.2 ppb for SO_2_ and 83.5 ppb for CO

In the adjusted model, data were adjusted for sex (categorical, for total sample only), age (continuous), BMI (continuous), smoking status (categorical), daily alcohol intake (categorical), physical activity (categorical), diabetes mellitus (categorical), hypertension (categorical), dyslipidemia (categorical), season (categorical), and temperature (continuous).

Bold values indicate statistically significant associations (P < 0.05).

**Fig. 2:**
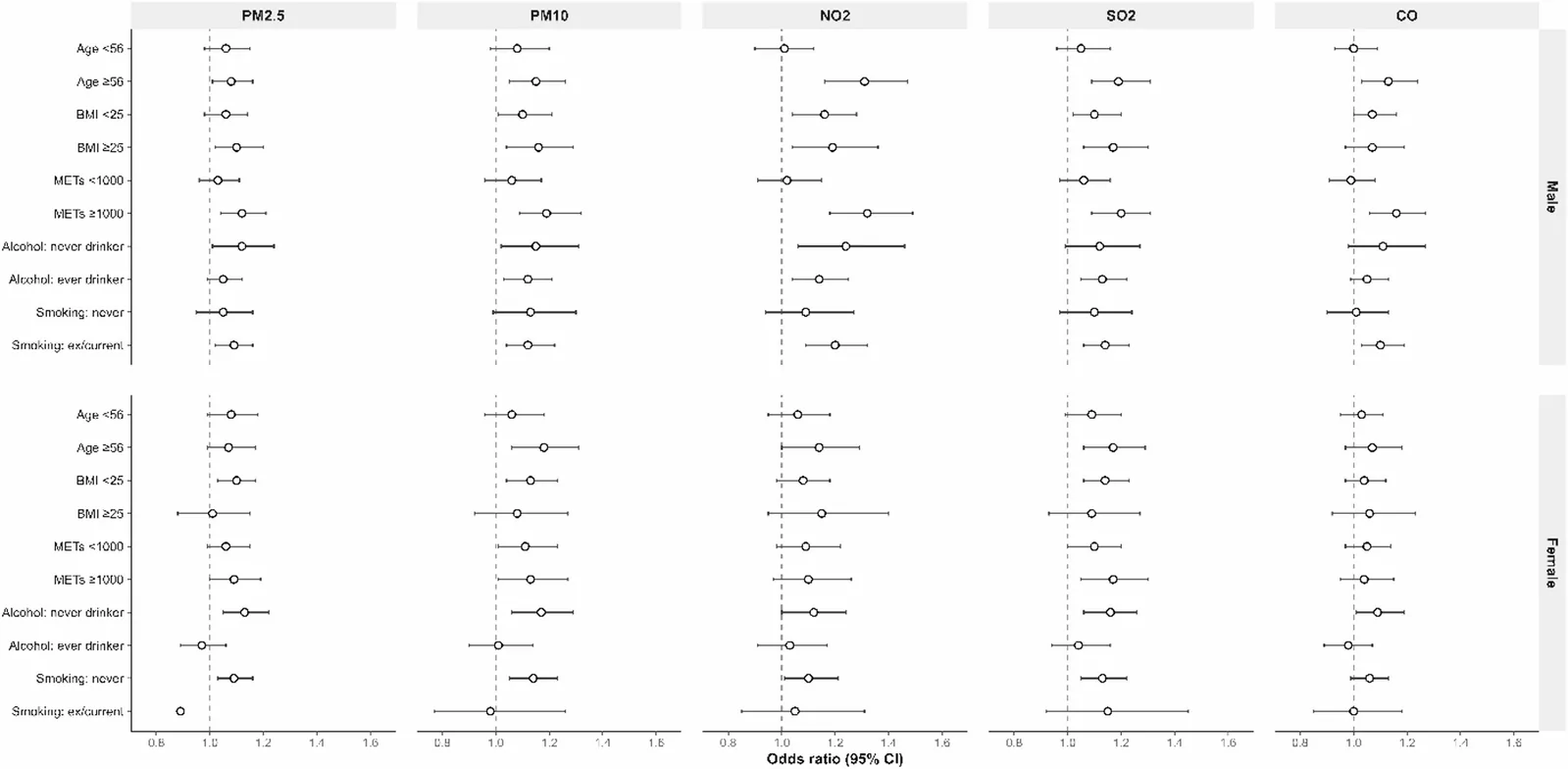
Subgroup analysis of the associations between annual air pollution exposure and dichotomized TSH. Forest plots presenting subgroup analyses of annual exposure to air pollutants with thyroid function: the highest quartile of TSH (Q4 vs. Q1–3) in males and females. Data were adjusted for age (continuous), BMI (continuous), smoking status (categorical), daily alcohol intake (categorical), physical activity (categorical), diabetes mellitus (categorical), hypertension (categorical), dyslipidemia (categorical), season (categorical), and temperature (continuous). TSH, thyrotropin; FT4, free thyroxine; BMI, body mass index; METs, Metabolic Equivalent Task minutes per week. PM_2.5_, Annual average air pollution exposure levels of particulate matter ≤ 2.5 μm in diameter; PM_10_, Annual average air pollution exposure levels of particulate matter ≤ 10 μm in diameter; NO_2_, Annual average air pollution exposure levels of nitrogen dioxide; SO_2_, Annual average air pollution exposure levels of sulfur dioxide; CO, Annual average air pollution exposure levels of carbon monoxide. The *X*-axis represents odds ratios with 95% confidence intervals

**Fig. 3:**
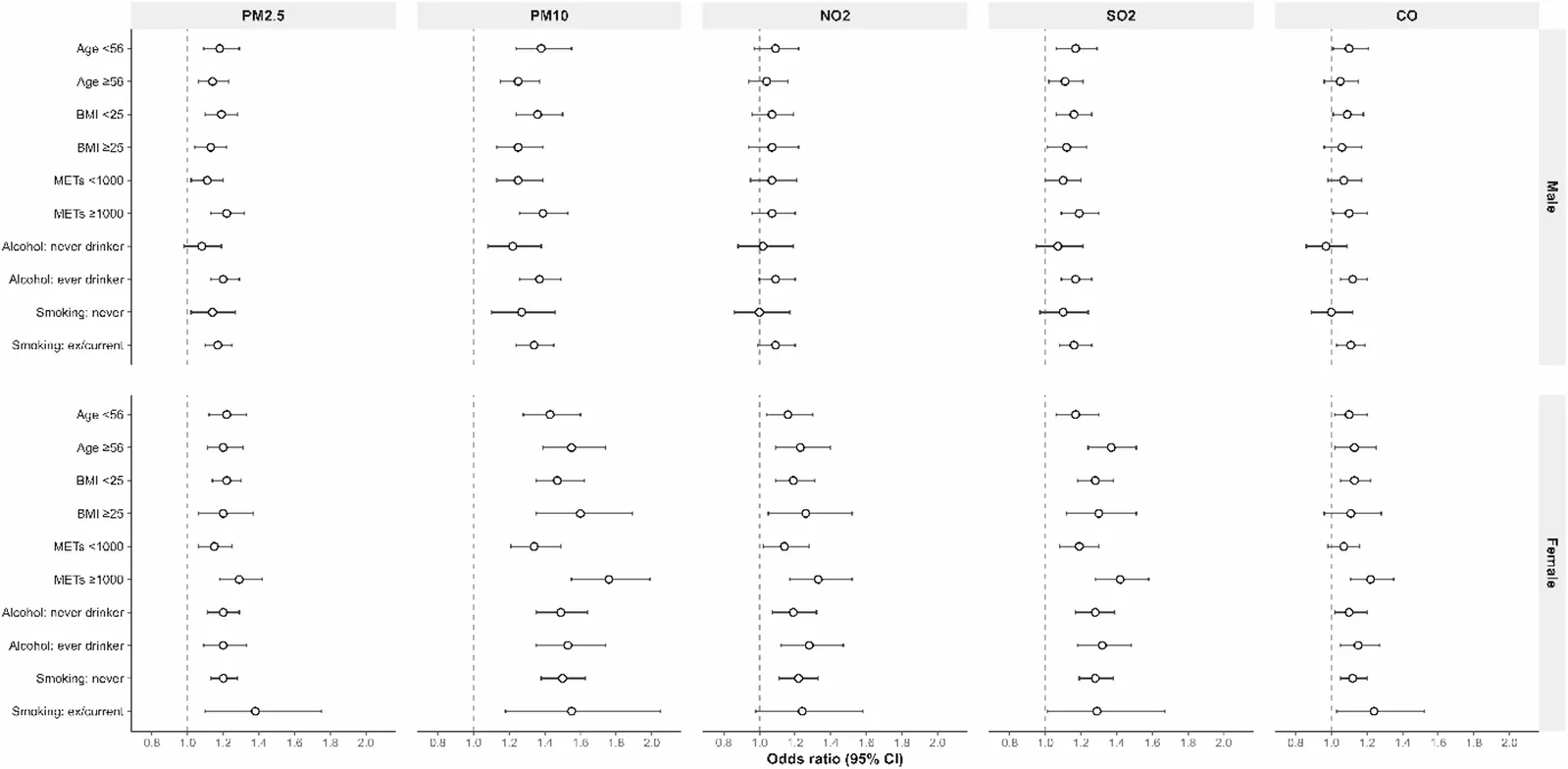
Subgroup analysis of the associations between annual air pollution exposure and dichotomized FT4 Levels. Forest plots presenting subgroup analyses of annual exposure to air pollutants with thyroid function: the lowest quartile of FT4 (Q1 vs. Q2–4) in males and females. Data were adjusted for age (continuous), BMI (continuous), smoking status (categorical), daily alcohol intake (categorical), physical activity (categorical), diabetes mellitus (categorical), hypertension (categorical), dyslipidemia (categorical), season (categorical), and temperature (continuous). TSH, thyrotropin; FT4, free thyroxine; BMI, body mass index; METs, Metabolic Equivalent Task minutes per week. PM_2.5_, Annual average air pollution exposure levels of particulate matter ≤ 2.5 μm in diameter; PM_10_, Annual average air pollution exposure levels of particulate matter ≤ 10 μm in diameter; NO_2_, Annual average air pollution exposure levels of nitrogen dioxide; SO_2_, Annual average air pollution exposure levels of sulfur dioxide; CO, Annual average air pollution exposure levels of carbon monoxide. The *X*-axis represents odds ratios with 95% confidence intervals

Figure 2 and 3 shows the results of the stratified logistic regression analyses in subgroups by age (median 56), BMI (25 kg/m^2^), 1,000 MET minutes per week, alcohol intake, and smoking status. P-values for interactions were shown in Supplementary Tables 2 and Supplementary Table 3. Overall, the results of the associations in each subgroup from the stratified analysis did not differ from those of the overall sample. In stratified analyses, statistically significant effect modification was observed for age in the association between NO₂ exposure and the high TSH group among males, for alcohol consumption in the association between PM_2.5_ exposure and low FT4 among males, and for physical activity in the association of PM_2.5_, PM_10_, and SO₂ with low FT4 among females.

In sensitivity analysis excluding low TSH/high FT4, several associations with log-transformed TSH were attenuated and no longer statistically significant in the total population (Supplementary Table 4). In particular, PM_2.5_ and PM_10_ were not significantly associated with log-transformed TSH after adjustment, whereas NO₂ retained a positive association with log-transformed TSH (β = 0.030, *p* = 0.005). In sex-stratified analyses, a significant positive association between NO₂ and log-transformed TSH persisted among males (β = 0.043, *p* = 0.002), while no significant associations were observed among females. For log-transformed FT4, inverse associations with air pollutant exposure remained and were statistically significant across most pollutants in the total population (Supplementary Table 5). PM_2.5_, PM_10_, NO₂, SO₂, and CO all showed consistent negative associations with FT4 after adjustment. In sex-stratified analyses, these inverse associations were generally more pronounced and consistently significant among females, whereas non-significant associations were observed among males for NO_2_.

In multivariable-adjusted multinomial logistic regression analyses, using the low TSH/high FT4 group as the reference category, higher annual exposure to air pollutants was positively associated with the high TSH–high FT4, low TSH–low FT4, and high TSH–low FT4 groups, with the strongest association observed for the high TSH/low FT4 group (Table 4). In sex-stratified analyses, larger effect estimates were shown in females.

**Table 4:** Multinomial logistic regression of the associations between annual air pollution exposure and four categories of thyroid function

Air pollutant exposures	Total (*n* = 16,193)				Male (*n* = 8,812)				Female (*n* = 7,381)			
	Unadjusted		Adjusted		Unadjusted		Adjusted		Unadjusted		Adjusted	
	OR (95% CI)	*p*	OR (95% CI)	*p*	OR (95% CI)	*p*	OR (95% CI)	*p*	OR (95% CI)	*p*	OR (95% CI)	*p*
*PM*_2.5_ (*µg/m*^*3*^)												
Low TSH-high FT4	Reference		Reference		Reference		Reference		Reference		Reference	
High TSH-high FT4	**1.05 (1.00-1.10)**	**0.044**	**1.06 (1.01–1.11)**	**0.021**	**1.07 (1.00-1.13)**	**0.046**	**1.07 (1.01–1.14)**	**0.031**	1.03 (0.96–1.10)	0.406	1.04 (0.97–1.12)	0.276
Low TSH-low FT4	**1.15 (1.09–1.20)**	**< 0.001**	**1.17 (1.12–1.23)**	**< 0.001**	**1.13 (1.07–1.21)**	**< 0.001**	**1.17 (1.09–1.24)**	**< 0.001**	**1.16 (1.08–1.25)**	**< 0.001**	**1.18 (1.10–1.27)**	**< 0.001**
High TSH-lowFT4	**1.22 (1.15–1.31)**	**< 0.001**	**1.25 (1.17–1.34)**	**< 0.001**	**1.18 (1.08–1.29)**	**< 0.001**	**1.22 (1.11–1.33)**	**< 0.001**	**1.27 (1.15–1.39)**	**< 0.001**	**1.30 (1.18–1.43)**	**< 0.001**
*PM*_*10*_ *(µg/m*^*3*^*)*												
Low TSH-high FT4	Reference		Reference		Reference		Reference		Reference		Reference	
High TSH-high FT4	**1.07 (1.01–1.14)**	**0.020**	**1.08 (1.02–1.15)**	**0.011**	**1.10 (1.01–1.18)**	**0.022**	**1.10 (1.02–1.19)**	**0.018**	1.04 (0.95–1.14)	0.358	1.05 (0.96–1.15)	0.269
Low TSH-low FT4	**1.32 (1.24–1.40)**	**< 0.001**	**1.37 (1.29–1.46)**	**< 0.001**	**1.24 (1.15–1.35)**	**< 0.001**	**1.31 (1.21–1.42)**	**< 0.001**	**1.42 (1.30–1.56)**	**< 0.001**	**1.45 (1.32–1.60)**	**< 0.001**
High TSH-lowFT4	**1.47 (1.35–1.60)**	**< 0.001**	**1.55 (1.42–1.69)**	**< 0.001**	**1.36 (1.21–1.52)**	**< 0.001**	**1.45 (1.29–1.64)**	**< 0.001**	**1.61 (1.42–1.83)**	**< 0.001**	**1.68 (1.48–1.91)**	**< 0.001**
*NO*_*2*_ *(ppb)*												
Low TSH-high FT4	Reference		Reference		Reference		Reference		Reference		Reference	
High TSH-high FT4	1.05 (0.98–1.13)	0.132	1.07 (1.00-1.15)	0.058	1.09 (0.99–1.20)	0.064	1.10 (1.00-1.22)	0.044	1.01 (0.92–1.11)	0.837	1.03 (0.93–1.14)	0.578
Low TSH-low FT4	1.02 (0.96–1.09)	0.532	1.07 (1.00-1.15)	0.059	0.95 (0.87–1.04)	0.291	1.01 (0.91–1.11)	0.915	**1.12 (1.01–1.23)**	**0.029**	**1.16 (1.04–1.28)**	**0.005**
High TSH-lowFT4	**1.25 (1.14–1.38)**	**< 0.001**	**1.36 (1.23–1.50)**	**< 0.001**	**1.21 (1.06–1.38)**	**0.005**	**1.33 (1.16–1.53)**	**< 0.001**	**1.30 (1.13–1.50)**	**< 0.001**	**1.39 (1.21–1.60)**	**< 0.001**
*SO*_*2*_ *(ppb)*												
Low TSH-high FT4	Reference		Reference		Reference		Reference		Reference		Reference	
High TSH-high FT4	**1.07 (1.02–1.13)**	**0.010**	**1.09 (1.03–1.15)**	**0.004**	**1.09 (1.01–1.17)**	**0.029**	**1.09 (1.01–1.18)**	**0.020**	1.06 (0.98–1.15)	0.159	1.07 (0.99–1.17)	0.088
Low TSH-low FT4	**1.13 (1.07–1.20)**	**< 0.001**	**1.16 (1.10–1.23)**	**< 0.001**	1.06 (0.99–1.14)	0.097	1.11 (1.03–1.20)	0.007	**1.23 (1.13–1.33)**	**< 0.001**	**1.23 (1.14–1.34)**	**< 0.001**
High TSH-lowFT4	**1.31 (1.21–1.41)**	**< 0.001**	**1.38 (1.28–1.49)**	**< 0.001**	**1.23 (1.11–1.37)**	**< 0.001**	**1.32 (1.19–1.47)**	**< 0.001**	**1.40 (1.25–1.57)**	**< 0.001**	**1.46 (1.30–1.63)**	**< 0.001**
*CO (ppb)*												
Low TSH-high FT4	Reference		Reference		Reference		Reference		Reference		Reference	
High TSH-high FT4	1.01 (0.96–1.06)	0.830	1.01(0.97–1.06)	0.598	1.02 (0.95–1.09)	0.667	1.02 (0.96–1.09)	0.503	0.99 (0.93–1.07)	0.888	1.00 (0.93–1.08)	0.917
Low TSH-low FT4	1.02 (0.97–1.07)	0.475	**1.05 (1.00-1.10)**	**0.031**	0.99 (0.92–1.06)	0.746	1.03 (0.97–1.10)	0.364	1.06 (0.98–1.14)	0.132	**1.09 (1.01–1.17)**	**0.026**
High TSH-lowFT4	**1.16 (1.08–1.25)**	**< 0.001**	**1.23 (1.15–1.31)**	**< 0.001**	**1.14 (1.03–1.27)**	**0.011**	**1.23 (1.13–1.35)**	**< 0.001**	**1.18 (1.06–1.30)**	**0.002**	**1.23 (1.13–1.34)**	**< 0.001**

TSH, thyrotropin; FT4, free thyroxine; PM_2.5_, particulate matter ≤ 2.5 μm in diameter; PM_10_, particulate matter ≤ 10 μm in diameter; NO_2_, nitrogen dioxide; SO_2_, sulfur dioxide; CO, carbon monoxide; OR, odds ratio; CI, confidence interval

The ORs and 95% CIs of air pollutants were scaled to the interquartile range (IQR) for each air pollutant: 2.7 µg/m^3^ for PM_2.5_, 7.4 µg/m^3^ for PM_10_, 14.1 ppb for NO_2_, 1.2 ppb for SO_2_ and 83.5 ppb for CO

High TSH was defined as TSH ≥ 1.96 mIU/L in males and ≥ 2.26 mIU/L in females, and low TSH was defined as TSH < 1.96 mIU/L in males and < 2.26 mIU/L in females. High FT4 was defined as FT4 ≥ 1.18 mIU/L in males and ≥ 1.14 mIU/L in females, and low FT4 was defined as FT4 < 1.18 mIU/L in males and < 1.14 mIU/L in females

In the adjusted model, data were adjusted for sex (categorical, for total sample only), age (continuous), BMI (continuous), smoking status (categorical), daily alcohol intake (categorical), physical activity (categorical), diabetes mellitus (categorical), hypertension (categorical), dyslipidemia (categorical), season (categorical), and temperature (continuous).

Bold values indicate statistically significant associations (P < 0.05).

## Discussion

In this large population-based cohort study, all five air pollutants (PM_2.5_, PM_10_, NO_2_, SO_2_, and CO) showed significant positive associations with serum TSH levels and negative associations with FT4 levels. Significant non-linear associations were observed for PM_2.5_ and NO₂ with log-transformed TSH, whereas all air pollutants showed non-linear associations with log-transformed FT4. Annual exposures to PM_2.5_, PM_10_, NO_2_, SO_2_, and CO were significantly associated with the highest quartile of TSH and the lowest quartile of FT4 levels, respectively. There were no substantial differences in the associations between air pollution exposure and thyroid function across various subgroups based on demographic and lifestyle factors. Higher annual average concentrations of air pollutants were positively associated with the high TSH–low FT4 group. These associations were consistently observed across particulate and gaseous pollutants, with the largest effect estimates observed for PM_10_, and the effect estimates were generally larger in females.

The positive associations with TSH levels and negative associations with FT4 are in line with previous studies showing that air pollutant exposures affect thyroid function in vulnerable populations, including pregnant females and newborns [32, 33], as well as in the general adult population [34, 35]. Our team has previously reported the significant associations of NO_2_ and CO exposures with TSH levels and FT4 levels respectively in 4,704 Korean adults [15]. After stratification by sex, positive associations between PM_10_ exposure and TSH levels became significant in males. The study population included 3,450 males and 1,244 females, and PM_2.5_ exposure was not included in the analysis due to limited measurements by conventional methods. A recent cross-sectional study of 5,626 participants from the Korea National Health and Nutrition Examination Survey found that short-term (7-day) exposures to NO_2_ and CO were positively associated with TSH levels, but not with PM_2.5_ and _2_SO_2_. In addition, exposures to PM_2.5_ were negatively associated with FT4 levels [36]. Another cross-sectional study of 3,859 Spanish adults with TSH levels of 0.1-20.0mIU/L showed PM_2.5_ levels corresponding to the year of health examination were associated with high TSH (> 95th percentile) and low FT4 (≤ 5th percentile), respectively [17]. In the linear regression models of the study, PM_2.5_ levels showed a significant association with FT4, but not with TSH levels. Another pollutant in the study, NO_2_, did not show significant associations with thyroid function traits. A large population-based study of 327,913 Chinese adults found a negative association between PM_2.5_ exposure and serum FT4 levels, with no significant association with TSH [34]. However, because only age, sex, ambient temperature, and season were included as covariates in the multivariable models, residual confounding factors cannot be excluded and may have biased the observed associations toward the null. These differences in findings could further be explained by the study population, the average levels of individual pollutants, and the adjusted covariates in the models. Although the significance varied across studies, consistently significant associations with FT4 were demonstrated, whereas inconsistent associations were shown in the case of TSH. In contrast, our findings demonstrate consistently significant associations not only with FT4 but also with TSH. Furthermore, our results suggest that air pollutant exposure may be associated with biologically meaningful shifts in thyroid function parameters, rather than only changes that reach clinically defined thresholds.

Although the underlying mechanism for the link between air pollution and thyroid function has not been fully explored, several possible pathways can be proposed based on the available evidence. First, air pollution-mediated systemic effects, including oxidative stress and inflammatory responses, could be possible explanations [37, 38]. The disruption of the thyroid gland by oxidative stress and the hypothalamic-pituitary-thyroid axis through inflammatory markers, such as interleukin-6, has been reported to inhibit thyroxine activation [39]. In line with this, lower thyroid hormone levels may be perceived as compensatory mechanisms to counteract oxidative stress induced by air pollutant exposure. In addition, the sensitivity of the hypothalamic-pituitary-thyroid axis might be affected by air pollution via the same mechanism. Second, hepatic dysfunction induced by exposure to air pollutants has been associated with reduced transthyretin expression, a carrier protein for FT4 [40]. This disruption impairs the transport of FT4 in plasma and disrupts thyroid hormone homeostasis. Third, PM_2.5_ also decreases thyroid peroxidase levels, thereby inhibiting iodine activation and leading to reduced circulating FT4 levels with elevated TSH levels [41]. Finally, the experimental data showed that air pollution disrupts estrogen receptor signaling, one of the crucial nuclear receptors involved in regulating thyroid hormones, which can contribute to these associations [42].

There are several strengths that support our findings. The estimation of annual air pollution exposures was based on validated modeling techniques to ensure reliable exposure assessment. Because TSH and FT4 are essential indicators of thyroid function parameters, we examined the association with four categories combining TSH and FT4. Another strength of this study is its comprehensive adjustment for various lifestyle and clinical factors that may influence thyroid function. Furthermore, as most evidence suggests that PM_2.5_ is the principal air pollutant with the biggest threat to public health, this study also included PM_2.5_ in the estimations [43]. There are some limitations to be cautious in interpretation. The cross-sectional design limits the establishment of causal relationships. This study assessed thyroid function at a single time, which may not fully capture the longitudinal effects of air pollution exposures on thyroid function. Furthermore, potential confounding factors such as occupational exposure, indoor exposure, and outdoor activity-related exposure were not available due to data limitations. The absence of thyroid ultrasound and thyroid autoantibody data limits the ability to distinguish underlying etiologies of thyroid dysfunction. Despite those participants with self-reported previous diagnosis of thyroid disorders were excluded, information on medication use, including levothyroxine and anti-thyroid drugs, was not available, which may have influenced thyroid hormone levels. The lack of information on individual iodine status, specific medication use (amiodarone and glucocorticoids), dietary supplement use, or family history of thyroid disorder and autoimmune disease could introduce potential confounding effects. Thyroid disorders are often asymptomatic and may be incidentally detected during routine health examinations [44]. Accordingly, misclassification of thyroid disease status cannot be excluded. Different thyroid function profiles may represent distinct clinical entities with heterogeneous etiologies, and therefore, the observed associations should be interpreted with caution. A future longitudinal study with a longer study period with repeated air pollutant and thyroid function parameter measurements, as well as detailed clinical information, including family history of thyroid disease, is warranted to further elucidate these associations.

## Conclusions

In conclusion, this study provides epidemiologic evidence that annual air pollutant exposure is positively associated with TSH and negatively associated with FT4 levels. Annual air pollutant exposure is associated with alterations in thyroid function, indicating a possible marker of thyroid dysfunction. These findings highlight the potential endocrine-disrupting effects of air pollution and emphasize the necessity for targeted public health interventions.

## Supplementary Information

Below is the link to the electronic supplementary material.

Supplementary Material 1

Supplementary Material 2

## Data Availability

Data supporting the findings of this study are not publicly available and are available on request from the corresponding authors, Hyun Jin Kim and Jin Ho Park.

## Abbreviations

TSH: Thyroid-stimulating hormone
FT4: Free thyroxine
PM_2.5_: Particulate matter with diameters of ≤ 2.5 μm
PM_10_: Particulate matter with diameters of ≤ 10 μm
SO₂: Sulfur dioxide
CO: Carbon monoxide
CMAQ: Community Multiscale Air Quality
MET: Metabolic equivalent
IPAQ: International Physical Activity Questionnaire
HbA1c: Hemoglobin A1c
BP: Blood pressure
LDL: Low-density lipoprotein
