# Supplementary material for: Association between annual air pollutant exposure and thyroid function: a retrospective cross-sectional study
Source: J Endocrinol Invest. 2026 Aug 29;49(10):2679–91. doi: 10.1007/s40618-026-02965-6 (PMC13627222; doi:10.1007/s40618-026-02965-6)
Supplement: Supplementary file 1 [file 40618_2026_2965_MOESM1_ESM.docx]

Supplementary Table 1. Univariate analysis of covariates with thyroid function

|  | **Log TSH** | | **Log FT4** | |
| --- | --- | --- | --- | --- |
|  | *β* (95% CI) | *p*-value | *β* (95% CI) | *p*-value |
| Age (years) | 0.000 (0.000, 0.001) | 0.160 | **-0.001 (-0.001, -0.001)** | **<0.001** |
| BMI (kg/m^2^) | -0.001 (-0.003, 0.002) | 0.567 | **-0.001 (-0.002, -0.001)** | **<0.001** |
| Physical activity  (MET minutes per week) |  |  |  |  |
| METs <500 | Reference |  | Reference |  |
| 500<METs <1000 | **0.048(0.021,0.075)** | **<0.001** | 0.000(-0.006,0.007) | 0.914 |
| METs≥1000 | -0.012(-0.003,0.006) | 0.202 | -0.001(-0.006,0.003) | 0.569 |
| Alcohol intake |  |  |  |  |
| None | Reference |  | Reference |  |
| Less than 2 drinks/day | **-0.043(-0.062, -0.024)** | **<0.001** | **0.026(0.022,0.031)** | **<0.001** |
| More than 2 drinks/day | **-0.095(-0.120, -0.071)** | **<0.001** | **0.056(0.049,0.062)** | **<0.001** |
| Smoking habit |  |  |  |  |
| Never smoker | Reference |  | Reference |  |
| Ex-smoker | **-0.041(-0.061, -0.021)** | **<0.001** | **0.030(0.025,0.035)** | **<0.001** |
| Current smoker | **-0.156(-0.179, -0.133)** | **<0.001** | **0.041(0.035,0.047)** | **<0.001** |
| Season |  |  |  |  |
| Spring | Reference |  | Reference |  |
| Summer | **-0.037 (-0.061, -0.013)** | **0.003** | **-0.015 (-0.021, -0.009)** | **<0.001** |
| Autumn | 0.006 (-0.018, 0.031) | 0.616 | -0.002 (-0.008, 0.004) | 0.562 |
| Winter | **0.066 (0.042, 0.090)** | **<0.001** | **-0.011 (-0.017, -0.005)** | **<0.001** |
| Temperature (°C) | **-0.003 (-0.004, -0.002)** | **<0.001** | 0.000 (0.000, 0.000) | 0.867 |
| Hypertension |  |  |  |  |
| No | Reference |  | Reference |  |
| Yes | **-0.022 (-0.040, -0.004)** | **0.017** | 0.000 (-0.005, 0.004) | 0.853 |
| Diabetes mellitus |  |  |  |  |
| No | Reference |  | Reference |  |
| Yes | **-0.036 (-0.061, -0.011)** | **0.005** | 0.003 (-0.003, 0.009) | 0.343 |
| Dyslipidemia |  |  |  |  |
| No | Reference |  | Reference |  |
| Yes | -0.002 (-0.019, 0.015) | 0.776 | 0.001 (-0.003, 0.006) | 0.497 |

Abbreviations: TSH, thyrotropin; FT4, free thyroxine; MET, Metabolic Equivalent Task; BMI, body mass index; CI, confidence interval

Supplementary Table 2. Subgroup analysis of the associations between annual air pollution exposure and dichotomized TSH

|  | Male | | | | Female | | | |
| --- | --- | --- | --- | --- | --- | --- | --- | --- |
| High TSH | OR | 95%CI | p-value | Pint | OR | 95%CI | p-value | Pint |
| PM_2.5_ |  |  |  |  |  |  |  |  |
| Age |  |  |  |  |  |  |  |  |
| <56 | 1.06 | (0.98-1.15) | 0.141 | 0.697 | 1.08 | (0.99-1.18) | 0.080 | 0.624 |
| ≥56 | **1.08** | **(1.01-1.16)** | **0.035** |  | 1.07 | (0.99-1.17) | 0.093 |  |
| BMI |  |  |  |  |  |  |  |  |
| <25 | 1.06 | (0.98-1.14) | 0.128 | 0.252 | **1.10** | **(1.03-1.17)** | **0.006** | 0.583 |
| ≥25 | **1.10** | **(1.02-1.20)** | **0.020** |  | 1.01 | (0.88-1.15) | 0.929 |  |
| METs | |  |  |  |  |  |  |  |
| <1000 | 1.03 | (0.96-1.11) | 0.422 | 0.314 | 1.06 | (0.99-1.15) | 0.116 | 0.764 |
| ≥1000 | **1.12** | **(1.04-1.21)** | **0.003** |  | 1.09 | (1.00-1.19) | 0.065 |  |
| Alcohol |  |  |  |  |  |  |  |  |
| Never drinker | 1.12 | (1.01-1.24) | 0.026 | 0.263 | **1.13** | **(1.05-1.22)** | **0.001** | **0.026** |
| Ever drinker | 1.05 | (0.99-1.12) | 0.096 |  | 0.97 | (0.89-1.06) | 0.514 |  |
| Smoking |  |  |  |  |  |  |  |  |
| Never | 1.05 | (0.95-1.16) | 0.350 | 0.784 | **1.09** | **(1.03-1.16)** | **0.004** | 0.192 |
| Ex or Current | **1.09** | **(1.02-1.16)** | **0.011** |  | 0.89 | (0.73-1.10) | 0.284 |  |
| PM_10_ |  |  |  |  |  |  |  |  |
| Age |  |  |  |  |  |  |  |  |
| <56 | 1.08 | (0.98-1.20) | 0.128 | 0.206 | 1.06 | (0.96-1.18) | 0.245 | 0.320 |
| ≥56 | **1.15** | **(1.05-1.26)** | **0.003** |  | **1.18** | **(1.06-1.31)** | **0.003** |  |
| BMI |  |  |  |  |  |  |  |  |
| <25 | **1.10** | **(1.01-1.21)** | **0.031** | 0.349 | **1.13** | **(1.04-1.23)** | **0.004** | 0.784 |
| ≥25 | **1.16** | **(1.04-1.29)** | **0.007** |  | 1.08 | (0.92-1.27) | 0.361 |  |
| METs |  |  |  |  |  |  |  |  |
| <1000 | 1.06 | (0.96-1.17) | 0.273 | 0.193 | **1.11** | **(1.01-1.23)** | **0.034** | 0.814 |
| ≥1000 | **1.19** | **(1.09-1.32)** | **<0.001** |  | **1.13** | **(1.01-1.27)** | **0.038** |  |
| Alcohol |  |  |  |  |  |  |  |  |
| Never drinker | **1.15** | **(1.02-1.31)** | **0.025** | 0.969 | **1.17** | **(1.06-1.29)** | **0.001** | 0.086 |
| Ever drinker | **1.12** | **(1.03-1.21)** | **0.007** |  | 1.01 | (0.90-1.14) | 0.815 |  |
| Smoking |  |  |  |  |  |  |  |  |
| Never | 1.13 | (0.99-1.30) | 0.075 | 0.773 | **1.14** | **(1.05-1.23)** | **0.002** | 0.373 |
| Ex or Current | **1.12** | **(1.04-1.22)** | **0.004** |  | 0.98 | (0.77-1.26) | 0.886 |  |
| NO_2_ |  |  |  |  |  |  |  |  |
| Age |  |  |  |  |  |  |  |  |
| <56 | 1.01 | (0.90-1.12) | 0.909 | **0.030** | 1.06 | (0.95-1.18) | 0.333 | 0.544 |
| ≥56 | **1.31** | **(1.16-1.47)** | **<0.001** |  | **1.14** | **(1.00-1.29)** | **0.043** |  |
| BMI |  |  |  |  |  |  |  |  |
| <25 | **1.16** | **(1.04-1.28)** | **0.006** | 0.967 | 1.08 | (0.98-1.18) | 0.109 | 0.833 |
| ≥25 | **1.19** | **(1.04-1.36)** | **0.012** |  | 1.15 | (0.95-1.40) | 0.160 |  |
| METs |  |  |  |  |  |  |  |  |
| <1000 | 1.02 | (0.91-1.15) | 0.742 | 0.031 | 1.09 | (0.98-1.22) | 0.132 | 0.678 |
| ≥1000 | **1.32** | **(1.18-1.49)** | **<0.001** |  | 1.10 | (0.97-1.26) | 0.134 |  |
| Alcohol |  |  |  |  |  |  |  |  |
| Never drinker | **1.24** | **(1.06-1.46)** | **0.008** | 0.727 | **1.12** | **(1.00-1.24)** | **0.045** | 0.840 |
| Ever drinker | **1.14** | **(1.04-1.25)** | **0.005** |  | 1.03 | (0.91-1.17) | 0.670 |  |
| Smoking |  |  |  |  |  |  |  |  |
| Never | 1.09 | (0.94-1.27) | 0.272 | 0.350 | **1.10** | **(1.01-1.21)** | **0.033** | 0.817 |
| Ex or Current | **1.20** | **(1.09-1.32)** | **<0.001** |  | 1.05 | (0.85-1.31) | 0.647 |  |
| SO_2_ |  |  |  |  |  |  |  |  |
| Age |  |  |  |  |  |  |  |  |
| <56 | 1.05 | (0.96-1.16) | 0.271 | 0.217 | 1.09 | (0.99-1.20) | 0.099 | 0.502 |
| ≥56 | **1.19** | **(1.09-1.31)** | **<0.001** |  | **1.17** | **(1.06-1.29)** | **0.001** |  |
| BMI |  |  |  |  |  |  |  |  |
| <25 | 1.10 | (1.02-1.20) | 0.017 | 0.569 | **1.14** | **(1.06-1.23)** | **0.001** | 0.488 |
| ≥25 | **1.17** | **(1.06-1.30)** | **0.002** |  | 1.09 | (0.93-1.27) | 0.280 |  |
| METs |  |  |  |  |  |  |  |  |
| <1000 | 1.06 | (0.97-1.16) | 0.190 | 0.074 | **1.10** | **(1.00-1.20)** | **0.044** | 0.305 |
| ≥1000 | **1.20** | **(1.09-1.31)** | **<0.001** |  | **1.17** | **(1.05-1.30)** | **0.003** |  |
| Alcohol |  |  |  |  |  |  |  |  |
| Never drinker | 1.12 | (0.99-1.27) | 0.065 | 0.316 | **1.16** | **(1.06-1.26)** | **0.001** | 0.306 |
| Ever drinker | **1.13** | **(1.05-1.22)** | **0.001** |  | 1.04 | (0.94-1.16) | 0.437 |  |
| Smoking |  |  |  |  |  |  |  |  |
| Never | 1.10 | (0.97-1.24) | 0.130 | 0.734 | **1.13** | **(1.05-1.22)** | **0.001** | 0.944 |
| Ex or Current | **1.14** | **(1.06-1.23)** | **<0.001** |  | 1.15 | (0.92-1.45) | 0.233 |  |
| CO |  |  |  |  |  |  |  |  |
| Age |  |  |  |  |  |  |  |  |
| <56 | 1.00 | (0.93-1.09) | 0.917 | 0.412 | 1.03 | (0.95-1.11) | 0.452 | 0.733 |
| ≥56 | **1.13** | **(1.03-1.24)** | **0.009** |  | 1.07 | (0.97-1.18) | 0.172 |  |
| BMI |  |  |  |  |  |  |  |  |
| <25 | 1.07 | (1.00-1.16) | 0.067 | 0.233 | 1.04 | (0.97-1.12) | 0.222 | 0.520 |
| ≥25 | 1.07 | (0.97-1.19) | 0.188 |  | 1.06 | (0.92-1.23) | 0.442 |  |
| METs |  |  |  |  |  |  |  |  |
| <1000 | 0.99 | (0.91-1.08) | 0.835 | 0.101 | 1.05 | (0.97-1.14) | 0.237 | 0.633 |
| ≥1000 | **1.16** | **(1.06-1.27)** | **0.001** |  | 1.04 | (0.95-1.15) | 0.376 |  |
| Alcohol |  |  |  |  |  |  |  |  |
| Never drinker | 1.11 | (0.98-1.27) | 0.101 | 0.455 | **1.09** | **(1.01-1.19)** | **0.038** | 0.396 |
| Ever drinker | 1.05 | (0.99-1.13) | 0.130 |  | 0.98 | (0.89-1.07) | 0.587 |  |
| Smoking |  |  |  |  |  |  |  |  |
| Never | 1.01 | (0.90-1.13) | 0.918 | 0.309 | 1.06 | (0.99-1.13) | 0.099 | 0.986 |
| Ex or Current | **1.10** | **(1.03-1.19)** | **0.008** |  | 1.00 | (0.85-1.18) | 0.988 |  |

Abbreviations: TSH, thyrotropin; FT4, free thyroxine; BMI, body mass index; METs, Metabolic Equivalent Task minutes per week; PM_2.5_, particulate matter ≤ 2.5μm in diameter; PM_10_, particulate matter ≤ 10μm in diameter; NO_2_, nitrogen dioxide; SO_2_, sulfur dioxide; CO, carbon monoxide; OR, odds ratio; CI, confidence interval. Data were adjusted for sex (categorical, for total sample only), age (continuous), BMI (continuous), smoking status (categorical), daily alcohol intake (categorical), physical activity (categorical), diabetes mellitus (categorical), hypertension (categorical), dyslipidemia (categorical), season (categorical), and temperature (continuous).

Supplementary Table 3. Subgroup analysis of the associations between annual air pollution exposure and dichotomized FT4

|  | Male | | | | Female | | | |
| --- | --- | --- | --- | --- | --- | --- | --- | --- |
| Low FT4 | OR | 95%CI | p-value | Pint | OR | 95%CI | p-value | Pint |
| PM_2.5_ |  |  |  |  |  |  |  |  |
| Age |  |  |  |  |  |  |  |  |
| <56 | **1.18** | **(1.09-1.29)** | **<0.001** | 0.251 | **1.22** | **(1.12-1.33)** | **<0.001** | 0.224 |
| ≥56 | **1.14** | **(1.06-1.23)** | **<0.001** |  | **1.20** | **(1.11-1.31)** | **<0.001** |  |
| BMI |  |  |  |  |  |  |  |  |
| <25 | **1.19** | **(1.10-1.28)** | **<0.001** | 0.376 | **1.22** | **(1.14-1.30)** | **<0.001** | 0.264 |
| ≥25 | **1.13** | **(1.04-1.22)** | **0.004** |  | **1.20** | **(1.06-1.37)** | **0.004** |  |
| METs |  |  |  |  |  |  |  |  |
| <1000 | **1.11** | **(1.02-1.20)** | **0.013** | 0.062 | **1.15** | **(1.06-1.25)** | **<0.001** | **0.035** |
| ≥1000 | **1.22** | **(1.13-1.32)** | **<0.001** |  | **1.29** | **(1.18-1.42)** | **<0.001** |  |
| Alcohol |  |  |  |  |  |  |  |  |
| Never drinker | 1.08 | (0.98-1.19) | 0.130 | **0.020** | **1.20** | **(1.11-1.29)** | **<0.001** | 0.436 |
| Ever drinker | **1.20** | **(1.13-1.29)** | **<0.001** |  | **1.20** | **(1.09-1.33)** | **<0.001** |  |
| Smoking |  |  |  |  |  |  |  |  |
| Never | **1.14** | **(1.02-1.27)** | **0.019** | 0.358 | **1.20** | **(1.13-1.28)** | **<0.001** | 0.260 |
| Ex or Current | **1.17** | **(1.10-1.25)** | **<0.001** |  | **1.38** | **(1.10-1.75)** | **0.007** |  |
| PM_10_ |  |  |  |  |  |  |  |  |
| Age |  |  |  |  |  |  |  |  |
| <56 | **1.38** | **(1.24-1.55)** | **<0.001** | 0.362 | **1.43** | **(1.28-1.60)** | **<0.001** | 0.857 |
| ≥56 | **1.25** | **(1.15-1.37)** | **<0.001** |  | **1.55** | **(1.39-1.74)** | **<0.001** |  |
| BMI |  |  |  |  |  |  |  |  |
| <25 | **1.36** | **(1.24-1.50)** | **<0.001** | 0.097 | **1.47** | **(1.35-1.62)** | **<0.001** | 0.664 |
| ≥25 | **1.25** | **(1.13-1.39)** | **<0.001** |  | **1.60** | **(1.35-1.89)** | **<0.001** |  |
| METs |  |  |  |  |  |  |  |  |
| <1000 | **1.25** | **(1.13-1.39)** | **<0.001** | 0.088 | **1.34** | **(1.21-1.49)** | **<0.001** | **0.001** |
| ≥1000 | **1.39** | **(1.26-1.53)** | **<0.001** |  | **1.76** | **(1.55-1.99)** | **<0.001** |  |
| Alcohol |  |  |  |  |  |  |  |  |
| Never drinker | **1.22** | **(1.08-1.38)** | **0.001** | 0.117 | **1.49** | **(1.35-1.64)** | **<0.001** | 0.728 |
| Ever drinker | **1.37** | **(1.26-1.49)** | **<0.001** |  | **1.53** | **(1.35-1.74)** | **<0.001** |  |
| Smoking |  |  |  |  |  |  |  |  |
| Never | **1.27** | **(1.10-1.46)** | **0.001** | 0.721 | **1.50** | **(1.38-1.63)** | **<0.001** | 0.802 |
| Ex or Current | **1.34** | **(1.24-1.45)** | **<0.001** |  | **1.55** | **(1.18-2.05)** | **0.002** |  |
| NO_2_ |  |  |  |  |  |  |  |  |
| Age |  |  |  |  |  |  |  |  |
| <56 | 1.09 | (0.97-1.22) | 0.171 | 0.852 | **1.16** | **(1.04-1.30)** | **0.010** | 0.557 |
| ≥56 | 1.04 | (0.94-1.16) | 0.449 |  | **1.23** | **(1.09-1.40)** | **0.001** |  |
| BMI |  |  |  |  |  |  |  |  |
| <25 | 1.07 | (0.96-1.19) | 0.217 | 0.533 | **1.19** | **(1.09-1.31)** | **<0.001** | 0.641 |
| ≥25 | 1.07 | (0.94-1.22) | 0.306 |  | **1.26** | **(1.05-1.52)** | **0.015** |  |
| METs |  |  |  |  |  |  |  |  |
| <1000 | 1.07 | (0.95-1.21) | 0.266 | 0.731 | **1.14** | **(1.02-1.28)** | **0.024** | 0.088 |
| ≥1000 | 1.07 | (0.96-1.20) | 0.220 |  | **1.33** | **(1.17-1.52)** | **<0.001** |  |
| Alcohol |  |  |  |  |  |  |  |  |
| Never drinker | 1.02 | (0.88-1.19) | 0.781 | 0.465 | **1.19** | **(1.07-1.32)** | **0.002** | 0.498 |
| Ever drinker | 1.09 | (1.00-1.20) | 0.059 |  | **1.28** | **(1.12-1.47)** | **<0.001** |  |
| Smoking |  |  |  |  |  |  |  |  |
| Never | 1.00 | (0.86-1.17) | 0.993 | 0.709 | **1.22** | **(1.11-1.33)** | **<0.001** | 0.634 |
| Ex or Current | 1.09 | (0.99-1.20) | 0.068 |  | 1.24 | (0.98-1.58) | 0.082 |  |
| SO_2_ |  |  |  |  |  |  |  |  |
| Age |  |  |  |  |  |  |  |  |
| <56 | **1.17** | **(1.06-1.29)** | **0.002** | 0.441 | **1.17** | **(1.06-1.30)** | **0.002** | 0.133 |
| ≥56 | **1.11** | **(1.02-1.21)** | **0.014** |  | **1.37** | **(1.24-1.51)** | **<0.001** |  |
| BMI |  |  |  |  |  |  |  |  |
| <25 | **1.16** | **(1.06-1.26)** | **0.001** | 0.164 | **1.28** | **(1.18-1.38)** | **<0.001** | 0.278 |
| ≥25 | **1.12** | **(1.01-1.23)** | **0.030** |  | **1.30** | **(1.12-1.51)** | **0.001** |  |
| METs |  |  |  |  |  |  |  |  |
| <1000 | 1.10 | (1.00-1.20) | 0.056 | 0.139 | **1.19** | **(1.08-1.30)** | **<0.001** | **0.004** |
| ≥1000 | **1.19** | **(1.09-1.30)** | **<0.001** |  | **1.42** | **(1.28-1.58)** | **<0.001** |  |
| Alcohol |  |  |  |  |  |  |  |  |
| Never drinker | 1.07 | (0.95-1.21) | 0.244 | 0.053 | **1.28** | **(1.17-1.39)** | **<0.001** | 0.813 |
| Ever drinker | **1.17** | **(1.09-1.26)** | **<0.001** |  | **1.32** | **(1.18-1.48)** | **<0.001** |  |
| Smoking |  |  |  |  |  |  |  |  |
| Never | 1.10 | (0.97-1.24) | 0.155 | 0.840 | **1.28** | **(1.19-1.38)** | **<0.001** | 0.683 |
| Ex or Current | **1.16** | **(1.08-1.26)** | **<0.001** |  | **1.29** | **(1.01-1.67)** | **0.048** |  |
| CO |  |  |  |  |  |  |  |  |
| Age |  |  |  |  |  |  |  |  |
| <56 | **1.10** | **(1.01-1.21)** | **0.024** | 0.924 | **1.10** | **(1.02-1.20)** | **0.018** | 0.298 |
| ≥56 | 1.05 | (0.96-1.15) | 0.276 |  | **1.13** | **(1.02-1.25)** | **0.015** |  |
| BMI |  |  |  |  |  |  |  |  |
| <25 | **1.09** | **(1.01-1.18)** | **0.038** | 0.326 | **1.13** | **(1.05-1.22)** | **0.001** | 0.865 |
| ≥25 | 1.06 | (0.96-1.17) | 0.222 |  | 1.11 | (0.96-1.28) | 0.152 |  |
| METs |  |  |  |  |  |  |  |  |
| <1000 | 1.07 | (0.98-1.17) | 0.165 | 0.424 | 1.07 | (0.98-1.16) | 0.118 | 0.043 |
| ≥1000 | **1.10** | **(1.01-1.20)** | **0.033** |  | **1.22** | **(1.11-1.35)** | **<0.001** |  |
| Alcohol |  |  |  |  |  |  |  |  |
| Never drinker | 0.97 | (0.86-1.09) | 0.568 | 0.017 | **1.10** | **(1.02-1.20)** | **0.018** | 0.187 |
| Ever drinker | **1.12** | **(1.05-1.20)** | **0.001** |  | **1.15** | **(1.05-1.27)** | **0.004** |  |
| Smoking |  |  |  |  |  |  |  |  |
| Never | 1.00 | (0.89-1.12) | 0.964 | 0.994 | **1.12** | **(1.05-1.20)** | **0.001** | 0.602 |
| Ex or Current | **1.11** | **(1.03-1.19)** | **0.005** |  | **1.24** | **(1.03-1.52)** | **0.030** |  |

Abbreviations: TSH, thyrotropin; FT4, free thyroxine; BMI, body mass index; METs, Metabolic Equivalent Task minutes per week; PM_2.5_, particulate matter ≤ 2.5μm in diameter; PM_10_, particulate matter ≤ 10μm in diameter; NO_2_, nitrogen dioxide; SO_2_, sulfur dioxide; CO, carbon monoxide; OR, odds ratio; CI, confidence interval.

Data were adjusted for sex (categorical, for total sample only), age (continuous), BMI (continuous), smoking status (categorical), daily alcohol intake (categorical), physical activity (categorical), diabetes mellitus (categorical), hypertension (categorical), dyslipidemia (categorical), season (categorical), and temperature (continuous).

Supplementary Table 4. Sensitivity analysis of the association between annual air pollutant exposure and log-transformed TSH using linear regression after excluding participants with low TSH and high free T4.

| Air pollutants exposures | **Total (n = 6,806)** | | | | |  | **Male (n = 3,718)** | | | | |  | Female (n = 3,088) | | | | |
| --- | --- | --- | --- | --- | --- | --- | --- | --- | --- | --- | --- | --- | --- | --- | --- | --- | --- |
|  | Unadjusted | |  | Adjusted | |  | Unadjusted | |  | Adjusted | |  | Unadjusted | |  | Adjusted | |
|  | *β* (SE) | *p* |  | *β* (SE) | *p* |  | *β* (SE) | *p* |  | *β* (SE) | *p* |  | *β* (SE) | *p* |  | *β* (SE) | *p* |
| Log TSH |  |  |  |  |  |  |  |  |  |  |  |  |  |  |  |  |  |
| PM_2.5_ (μg/m^3^) | -0.008 (0.007) | 0.251 |  | -0.008 (0.007) | 0.251 |  | -0.009 (0.010) | 0.378 |  | -0.009 (0.010) | 0.380 |  | -0.010 (0.011) | 0.361 |  | -0.009 (0.011) | 0.443 |
| PM_10_ (μg/m^3^) | -0.013 (0.009) | 0.133 |  | -0.013 (0.009) | 0.146 |  | -0.005 (0.012) | 0.696 |  | -0.007 (0.012) | 0.558 |  | **-0.029 (0.013)** | **0.027** |  | -0.022 (0.013) | 0.098 |
| NO_2_ (ppb) | **0.032 (0.010)** | **0.002** |  | **0.030 (0.011)** | **0.005** |  | **0.045 (0.014)** | **0.001** |  | **0.043 (0.014)** | **0.002** |  | 0.006 (0.016) | 0.696 |  | 0.011 (0.016) | 0.483 |
| SO_2_ (ppb) | 0.009 (0.008) | 0.284 |  | 0.009 (0.008) | 0.284 |  | **0.022 (0.011)** | **0.046** |  | 0.020 (0.011) | 0.066 |  | -0.015 (0.012) | 0.231 |  | -0.006 (0.012) | 0.613 |
| CO (ppb) | 0.010 (0.008) | 0.178 |  | 0.008 (0.008) | 0.317 |  | 0.015 (0.010) | 0.141 |  | 0.014 (0.010) | 0.171 |  | -0.002 (0.012) | 0.834 |  | -0.001 (0.012) | 0.916 |
| Log FT4 |  |  |  |  |  |  |  |  |  |  |  |  |  |  |  |  |  |
| PM_2.5_ (μg/m^3^) | **-0.009 (0.002)** | **<0.001** |  | **-0.010 (0.002)** | **<0.001** |  | **-0.008 (0.002)** | **0.001** |  | **-0.010 (0.002)** | **<0.001** |  | **-0.008 (0.003)** | **0.004** |  | **-0.009 (0.003)** | **0.002** |
| PM_10_ (μg/m^3^) | **-0.016 (0.002)** | **<0.001** |  | **-0.017 (0.002)** | **<0.001** |  | **-0.012 (0.003)** | **<0.001** |  | **-0.016 (0.003)** | **<0.001** |  | **-0.019 (0.003)** | **<0.001** |  | **-0.020 (0.003)** | **<0.001** |
| NO_2_ (ppb) | **-0.006 (0.003)** | **0.020** |  | **-0.008 (0.003)** | **0.001** |  | 0.001 (0.004) | 0.850 |  | -0.004 (0.004) | 0.283 |  | **-0.011 (0.004)** | **0.005** |  | **-0.015 (0.004)** | **<0.001** |
| SO_2_ (ppb) | **-0.008 (0.002)** | **<0.001** |  | **-0.009 (0.002)** | **<0.001** |  | -0.003 (0.003) | 0.344 |  | **-0.006 (0.003)** | **0.045** |  | **-0.012 (0.003)** | **<0.001** |  | **-0.013 (0.003)** | **<0.001** |
| CO (ppb) | **-0.006 (0.002)** | **0.002** |  | **-0.008 (0.002)** | **<0.001** |  | -0.003 (0.003) | 0.320 |  | **-0.006 (0.003)** | **0.018** |  | **-0.008 (0.003)** | **0.005** |  | **-0.011 (0.003)** | **<0.001** |

Abbreviations: TSH, thyrotropin; FT4, free thyroxine; PM_2.5_, particulate matter ≤ 2.5μm in diameter; PM_10_, particulate matter ≤ 10μm in diameter; NO_2_, nitrogen dioxide; SO_2_, sulfur dioxide; CO, carbon monoxide; SE, standard error.

The beta coefficients (SE) of air pollutants were scaled to the interquartile range (IQR) for each air pollutant: 2.7μg/m3 for PM_2.5_, 7.4μg/m3 for PM_10_, 14.1ppb for NO_2_, 1.2ppb for SO_2_ and 83.5 ppb for CO.

In the adjusted model, data were adjusted for sex (categorical, for total sample only), age (continuous), BMI (continuous), smoking status (categorical), daily alcohol intake (categorical), physical activity (categorical), diabetes mellitus (categorical), hypertension (categorical), dyslipidemia (categorical), season (categorical), and temperature (continuous).

Supplementary Table 5. Sensitivity analysis of the association between annual air pollutant exposure and high TSH or low free T4 using logistic regression after excluding participants with low TSH and high free T4.

| Air pollutant exposures | **Total (n = 6,806)** | | | | |  | **Male (n = 3,718)** | | | | |  | **Female (n = 3,088)** | | | | | | | |
| --- | --- | --- | --- | --- | --- | --- | --- | --- | --- | --- | --- | --- | --- | --- | --- | --- | --- | --- | --- | --- |
|  | Unadjusted | |  | Adjusted | |  | Unadjusted | |  | Adjusted | |  | Unadjusted | |  | | Adjusted | | | |
|  | OR (95% CI) | *p* |  | OR (95% CI) | *p* |  | OR (95% CI) | *p* |  | OR (95% CI) | *p* |  | OR (95% CI) | *p* | |  | | OR (95% CI) | *p* |  |
| **High TSH (Quartile 4 versus Quartile 1-3)** | | | | | | | | | | | | | | | | | | | | |
| PM_2.5 (_μg/m^3^) | 0.96 (0.91-1.01) | 0.129 |  | 0.96 (0.90-1.01) | 0.108 |  | 0.97 (0.90-1.04) | 0.405 |  | 0.96 (0.89-1.03) | 0.280 |  | 0.94 (0.87-1.03) | 0.174 | |  | | 0.95 (0.87-1.03) | 0.222 |  |
| PM_10_ (μg/m^3^) | **0.90 (0.85-0.96)** | **0.002** |  | **0.90 (0.85-0.97)** | **0.003** |  | 0.94 (0.86-1.03) | 0.200 |  | 0.93 (0.84-1.01) | 0.101 |  | **0.85 (0.77-0.94)** | **0.002** | |  | | **0.87 (0.79-0.96)** | **0.006** |  |
| NO_2_ (ppb) | **1.09 (1.01-1.17)** | **0.033** |  | **1.08 (1.00-1.16)** | **0.044** |  | **1.18 (1.06-1.31)** | **0.002** |  | **1.18 (1.06-1.32)** | **0.003** |  | 0.98 (0.88-1.09) | 0.726 | |  | | 0.98 (0.88-1.10) | 0.766 |  |
| SO_2_ (ppb) | 1.01 (0.95-1.07) | 0.805 |  | 1.02 (0.95-1.08) | 0.629 |  | 1.06 (0.98-1.15) | 0.173 |  | 1.05 (0.96-1.14) | 0.259 |  | 0.95 (0.87-1.04) | 0.233 | |  | | 0.97 (0.89-1.07) | 0.547 |  |
| CO (ppb) | 1.03 (0.97-1.09) | 0.320 |  | 1.02 (0.97-1.08) | 0.459 |  | 1.06 (0.98-1.15) | 0.138 |  | 1.05 (0.97-1.14) | 0.216 |  | 0.99 (0.91-1.07) | 0.816 | |  | | 0.99 (0.91-1.07) | 0.745 |  |
| **Low FT4 (Quartile 1 versus Quartiles 2-4)** | | | | | | | | | | | | | | | | | | | | |
| PM_2.5 (_μg/m^3^) | **1.11 (1.05-1.18)** | **<0.001** |  | **1.13 (1.07-1.19)** | **<0.001** |  | **1.08 (1.00-1.16)** | **0.045** |  | **1.10 (1.02-1.18)** | **0.016** |  | **1.16 (1.07-1.26)** | **<0.001** | |  | | **1.17 (1.08-1.28)** | **<0.001** |  |
| PM_10_ (μg/m^3^) | **1.25 (1.17-1.33)** | **<0.001** |  | **1.27 (1.19-1.35)** | **<0.001** |  | **1.16 (1.06-1.26)** | **0.001** |  | **1.20 (1.10-1.32)** | **<0.001** |  | **1.37 (1.24-1.51)** | **<0.001** | |  | | **1.38 (1.25-1.53)** | **<0.001** |  |
| NO_2_ (ppb) | 1.03 (0.96-1.11) | 0.407 |  | 1.07 (0.99-1.15) | 0.095 |  | 0.94 (0.84-1.04) | 0.234 |  | 0.98 (0.88-1.09) | 0.720 |  | **1.15 (1.04-1.29)** | **0.010** | |  | | **1.19 (1.06-1.33)** | **0.003** |  |
| SO_2_ (ppb) | **1.10 (1.03-1.17)** | **0.002** |  | **1.11 (1.05-1.18)** | **0.001** |  | 1.02 (0.94-1.11) | 0.557 |  | 1.06 (0.97-1.15) | 0.196 |  | **1.20 (1.09-1.31)** | **<0.001** | |  | | **1.19 (1.09-1.31)** | **<0.001** |  |
| CO (ppb) | 1.05 (0.99-1.11) | 0.085 |  | **1.08 (1.02-1.14)** | **0.007** |  | 1.02 (0.94-1.10) | 0.700 |  | 1.06 (0.97-1.15) | 0.185 |  | **1.09 (1.01-1.18)** | **<0.001** | |  | | **1.12 (1.03-1.22)** | **0.007** |  |

Abbreviations: TSH, thyrotropin; FT4, free thyroxine; PM_2.5_, particulate matter ≤ 2.5μm in diameter; PM_10_, particulate matter ≤ 10μm in diameter; NO_2_, nitrogen dioxide; SO_2_, sulfur dioxide; CO, carbon monoxide; OR, odds ratio; CI, confidence interval.

The ORs and 95% CIs of air pollutants were scaled to the interquartile range (IQR) for each air pollutant: 2.7μg/m3 for PM_2.5_, 7.4μg/m3 for PM_10_, 14.1ppb for NO_2_, 1.2ppb for SO_2,_ and 83.5 ppb for CO. In the adjusted model, data were adjusted for sex (categorical, for total sample only), age (continuous), BMI (continuous), smoking status (categorical), daily alcohol intake (categorical), physical activity (categorical), diabetes mellitus (categorical), hypertension (categorical), dyslipidemia (categorical), season (categorical), and temperature (continuous).

**Supplementary Figure 1**. Flow Chart of Study Participants
